# The relationship between occupational physical activity and dyslipidaemia in farmers with varying working modes in southwest China: the China multi-ethnic cohort study

**DOI:** 10.1186/s12889-022-13266-x

**Published:** 2022-04-27

**Authors:** Lunwei Du, Feng Hong, Peng Luo, Ziyun Wang, Qibing Zeng, Han Guan, Haiyan Liu, Zhiping Yuan, Degan Xu, Fang Nie, Junhua Wang

**Affiliations:** 1 School of Public Health, the Key Laboratory of Environmental Pollution Monitoring and Disease Control, Ministry of Education, Guizhou Medical University, Guiyang, 550025 People’s Republic of China; 2 University Town of Hospital, Guizhou Medical University, Guiyang, 550025 People’s Republic of China; 3 Guiyang Center for Disease Control and Prevention, Guiyang, 550003 People’s Republic of China

**Keywords:** Cardiovascular disease, Dyslipidaemia, Farmers, Ethnic minorities, Occupational physical activity

## Abstract

**Background:**

Farmers are the integral members of rural communities. In the present study, we determined the association between occupational physical activity (OPA) of farmers and dyslipidaemia.

**Methods:**

We included 7649 farmers from The China Multi-Ethnic Cohort (CMEC) Study. The working modes of all farmers were divided into four types according to their self-reported seasonal changes in farming work and/or other job changes (1: no change; 2: changing job; 3: seasonal changes; and 4: seasonal and job changes). OPA was self-reported, and the OPA levels in the four groups were classified as Q1, Q2–Q3, and Q4 by quantile. Dyslipidaemia was defined as the presence of abnormalities in lipid indicators. Binary logistic regression was used to estimate the association between OPA and dyslipidaemia.

**Results:**

Compared with those in the no change group, the participants in other three groups were younger with lower level of education, annual income, and leisure-time physical activity (LTPA). Active OPA could reduce the risk of dyslipidaemia in the no change [men: odds ratios (OR) = 0.21, 95% confidence intervals (CI): 0.07–0.64; women: OR = 0.43, 95% CI: 0.20–0.93] and seasonal change (men: OR = 0.46, 95% CI: 0.27–0.77; women: OR = 0.59, 95% CI: 0.41–0.86) groups. However, in the seasonal and job change group (men: OR = 3.23, 95% CI: 1.06–9.80; women: OR = 3.24, 95% CI: 1.42–7.41), active OPA increased the risk of dyslipidaemia.

**Conclusions:**

Different OPA levels might lead to differences in association with blood lipid levels. Thus, OPA guidelines must be developed for farmers, especially for those who experience seasonal changes in farming work and job changes.

**Supplementary Information:**

The online version contains supplementary material available at 10.1186/s12889-022-13266-x.

## Background

Cardiovascular disease (CVD) is a major public health problem. According to the China cardiovascular report 2018, CVD was the main cause of death, with the mortality rate being higher in rural areas than in urban areas [1]. Dyslipidaemia, which is caused by an imbalance in any one of the four blood lipid indices, is one of the main causes of CVD. With the economic development, the blood lipid level and subsequently the cases of dyslipidaemia are increasing in China [2]. Dyslipidaemia is caused by many factors, including diet, economy, culture, physical inactivity, and obesity [3].

Physical activity (PA) is essential for healthy living. It can reduce the risk of many diseases [4]. Previous epidemiological studies have explored the relationship between PA and blood lipid levels [5–10]. However, the PA in these studies was total PA, and none of the studies have focused on the relationship between farmers’ PA and blood lipid levels. China is a large agricultural country [11]. OPA accounts for most of the activities of farmers. Studies have shown that not all types of OPA can improve health, and some types of OPA may be harmful [12]. In some rural areas of Guizhou Province, the OPA models of some farmers are different from those of other provinces. Specifically, on the one hand, Guizhou is the only province in China that has mountains without plains. Because of the special landform, automatic operation is not employed for agricultural production, and OPA accounts for a large proportion of all PAs. In addition, farming is an integral occupation of the rural population in Guizhou Province, and the activity pattern of farmers is seasonal. Consequently, some farmers’ production activities have been divided into farming and non-farming seasons, whereas some farmers’ production activities remain unchanged throughout the year. Typically, farmers perform moderate-to-high intensity, long-term planting, and harvesting activities during the farming season and perform less intensive agricultural activities in the non-farming season [13]. PA of the farmers who did not have seasonal changes in farming work was reported to fluctuate slightly, and no obvious change in PA was generally observed [13]. By contrast, due to differences in regions and economic levels, some farmers might also engage in other job profiles, such as construction workers and porters, to subsidise their families. These working modes have certain regional characteristics; this also causes such farmers to have various working modes, and the dose of OPA might increase. We do not know the impact of such a level on blood lipids. The questions that whether there is extra work for farming and non-farming seasons and what effect do OPA levels have on blood lipids in the people under these two combined working modes are worthy of in-depth discussion. In addition, the levels of OPA under different working modes and their impact on people’s health might vary.

Therefore, the present study evaluated the association between OPA of farmers and dyslipidaemia according to different working modes in the 30–79-year-old population of the China Multi-Ethnic Cohort (CMEC) Study.

## Methods

### Study participants

**Fig. 1:**
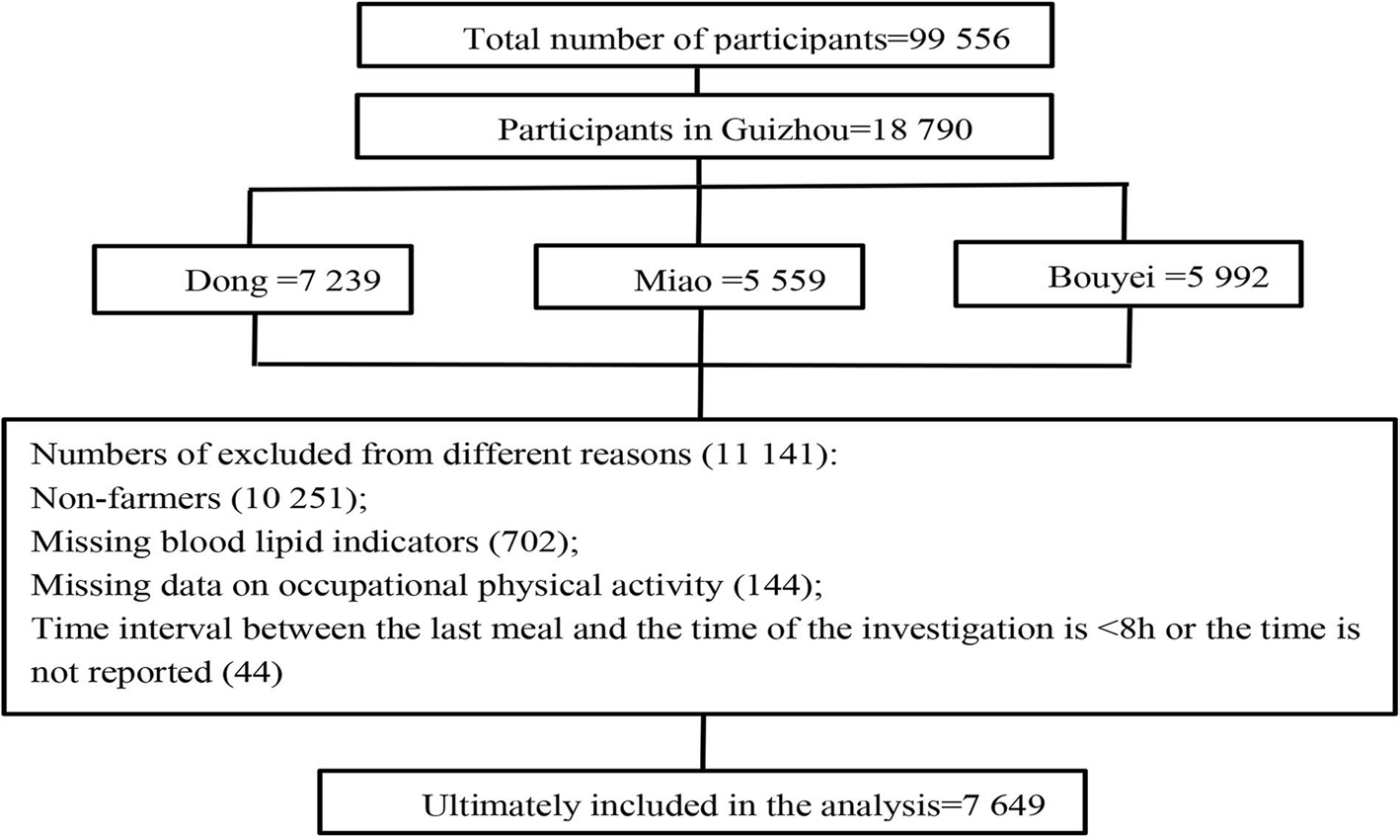
Flow chart of participants. *TC* Total cholesterol, *TG* Triglyceride, *LDL-C* Low-density lipoprotein cholesterol, *HDL-C* High-density lipoprotein cholesterol

Relevant data were obtained from the CMEC Study. The CMEC Study is a large-scale prospective cohort study. Related detailed information has been reported in the previous literature [14]. Briefly, we used a multi-stage stratified cluster sampling method to recruit participants from Congjiang county, Leishan county, and Liping county of Qiandongnan Miao and Dong Autonomous Prefecture and Guiding county, Libo county, and Dushan county of Qiannan Miao and Bouyei Autonomous Prefecture in Guizhou province, southwest China, from May 2018 to September 2019. We excluded some participants for various reasons (Fig. 1).

### Principal variables and covariates

PA was measured using a self-reported questionnaire. The questions were set according to the China Kadoorie Biobank (CKB) [15]. The questionnaire comprised four main aspects: occupational physical activity (OPA), transportation physical activity (TPA), domestic physical activity (DPA), and leisure-time physical activity (LTPA). The intensity level of each PA was determined by the updated compendium of PAs in 2011 [16]. The level of an individual’s daily PA was calculated by multiplying the specified metabolic equivalent tasks (METs) with the hours spent on the activity.

OPA during the past year was considered. Two sets of work-related questions, including nine questions about OPA, were asked to the farmers. However, for non-farmers, only one related question was asked: ‘In the past year, how active were you at work?’ Thus, our analysis was limited to the farmers in Dong, Miao, and Bouyei areas of Guizhou Province, wherein more detailed information about OPA was collected. Notably, the farmers in this study mainly refer to those engaged in agriculture and animal husbandry in the past year.

The following working mode-related questions were asked: 1) In the past 12 months, did your farming work change seasonally? 2) Apart from the agriculture work, did you have any other job? Accordingly, we divided the participants into four characteristic groups (1: no change group (no seasonal changes in farming work and no other jobs), 2: changing job group (only other jobs), 3: seasonal change group (seasonal changes in farming work), and 4: seasonal and job change group (seasonal changes in farming work and there were other jobs).

The covariates were collected through face-to-face interview by using the standard questionnaire; these covariates comprised sociodemographic characteristics (age, sex, ethnicity, education, marital status, and annual income) and lifestyle behaviours (drinking, smoking, tea intake, beverage intake, total energy intake, sleeping time, and leisure sedentary time).

Body mass index (BMI) was obtained from baseline measurements of weight and height by specially trained staff using calibrated instruments. It was calculated by dividing the measured weight (kg) by the height in metres square (m^2^).

### Timing of measurements

All the measurements (including data collection through self-reported questionnaire and face-to-face interviews and baseline measurements) were performed simultaneously throughout the day. The survey in our study was conducted from May 2018 to September 2019.

### Dyslipidaemia outcomes

The collected blood samples were sent to the JinYu Medical Laboratory Center of Guizhou Province for analysis of plasma high-density lipoprotein-cholesterol (HDL-C), low-density lipoprotein-cholesterol (LDL-C), total cholesterol (TC), and triglycerides (TG) levels after more than 8 h of fasting. Blood samples were collected in the morning, and the participants did not perform any exercise before blood collection. All the blood samples were analysed using the automatic biochemical instrument (Model: 7180, HITACHI, Japan). According to the Guideline for Prevention and Treatment of dyslipidaemia in China (2016 revised) [17], dyslipidaemia was defined as abnormality in any of the four indicators of blood lipids (TC ≥ 6.22 mmol/L, TG ≥ 2.26 mmol/L, LDL-C ≥ 4.14 mmol/L, and HDL-C < 1.04 mmol/L).

### Statistical analysis

Continuous variables (BMI, TG, TC, LDL-C, HDL-C, age, leisure sedentary time, sleeping time, and total energy intake) are expressed as median and interquartile range [M (P_25_, P_75_)] because they did not follow the normal distribution. The continuous variables were compared using the Kruskal–Wallis test. Categorical variables (OPA, TPA, DPA, LTPA, ethnicity, education, sex, smoking, drinking, annual income, married or cohabitating, tea intake, and beverage intake) are expressed in terms of percentage or constituent ratio. The categorical variables were compared using the chi-square test.

To compare the association between OPA in different working modes of farmers and dyslipidaemia, we divided the OPA under each working mode into three levels (Q1: ≤ P_25_, Q2–Q3: P_25_–P_75_, Q4: ≥ P_75_) by quartile. The results are as follows: no change group, P_25_ = 10.6 and P_75_ = 42.3 MET-hr/day; changing job group, P_25_ = 20.2 and P_75_ = 59.4 MET-hr/day; seasonal change group, P_25_ = 10.7 and P_75_ = 24.6 MET-hr/day; and seasonal and job changes group, P_25_ = 20.1 and P_75_ = 45.2 MET-hr/day. The adjusted odds ratios (ORs) and 95% confidence intervals (CIs) for each type of dyslipidaemia associated with OPA of farmers with different working modes were estimated using the binary logistic regression models, after adjusting for the confounding factors, which included age, sex, smoking, drinking, leisure sedentary time, sleeping time, total energy intake, married or cohabitating, beverage intake, BMI, TPA, DPA, LTPA, ethnicity (Dong, Miao, and Bouyei), education (no formal school, primary school, middle school, high school, and college or university), and annual income (< 12,000, 12,000–19,999, 20,000–59,999, 60,000–99,999, and ≥ 1,00,000 yuan/year). We used the directed acyclic graph (DAG) to identify the possible confounding factors (Figure S1). DAG was drawn by referring to the literature on the factors affecting OPA and dyslipidaemia (for more details, please refer to this website: http://www.dagitty.net/).

SPSS (version 22.0; SPSS Inc, Chicago, IL, USA) was used for data analysis. Statistical tests were two-sided, and a *P* value ≤ 0.05 was considered statistically significant.

## Results

### Characteristics of study participants

A total of 7649 participants were included in this study. The mean age of the participants was 52.7 years, and 63.4% participants were women. Overall, 14.0%, 5.5%, 61.1%, and 19.4% of the participants constituted the no change, changing job, seasonal change, and seasonal and job change groups, respectively. Generally, on an average, in the changing job, seasonal change, and seasonal and job change groups, younger men (more likely to be married men) had a lower level of education, LTPA, and annual income and a higher level of total energy intake than those in the no change group. In addition, the men in the three groups tended to smoke and drink more than those in the no change group (Table 1).

**Table 1:** Baseline characteristics of 7,649 study participants according to the baseline working mode

	**Total**	**No change**	**Changing job**	**Seasonal changes**	**Seasonal and job changes**	***P* value**
**Participants, *n*** **(%)**	7 649 (100.0)	1 074 (14.0)	417 (5.5)	4 671 (61.1)	1 487 (19.4)	
**Age (years)**	52.7 (46.1, 61.0)	56.2 (48.9, 65.1)	47.7 (41.3, 53.4)	54.0 (47.8, 62.3)	48.1 (42.6, 53.6)	< 0.001^*^
**BMI (kg/m**^**2**^)	23.7 (21.4, 26.2)	23.8 (21.3, 26.4)	24.4 (22.1, 26.7)	23.5 (21.2, 26.0)	24.0 (21.4, 26.2)	< 0.001^*^
**TC (mmol/L)**	4.9 (4.3, 5.5)	4.8 (4.3, 5.5)	4.9 (4.3, 5.4)	4.9 (4.3, 5.5)	4.8 (4.2,5.4)	0.016^*^
**TG (mmol/L)**	1.4 (1.0, 2.0)	1.4 (0.9, 2.0)	1.4 (1.0, 2.1)	1.4 (1.0, 2.0)	1.4 (1.0, 2.2)	0.002^*^
**LDL-C (mmol/L)**	2.7 (2.2, 3.2)	2.6 (2.2, 3.2)	2.7 (2.1, 3.2)	2.7 (2.2, 3.3)	2.6 (2.1, 3.1)	0.051^*^
**HDL-C (mmol/L)**	1.5 (1.3, 1.7)	1.5 (1.3, 1.7)	1.4 (1.2, 1.7)	1.5 (1.3, 1.7)	1.4 (1.2, 1.7)	< 0.001^*^
**Leisure sedentary time (h/week)**	14.0 (7.0, 21.0)	14.0 (7.0, 21.0)	14.0 (7.0, 21.0)	14.0 (7.0, 21.0)	14.00 (7.0, 21.0)	< 0.001^*^
**Total energy intake (kcal/week)**	10,447.9 (8121.3, 13,440.8)	10,110.5 (7847.2, 12,889.7)	11,132.3 (8651.5, 14,170.1)	10,235.32 (7950.2, 13,234.1)	11,349.2 (8886.3, 14,334.7)	< 0.001^*^
**Sleeping time (h/d)**	8.0 (6.0, 8.0)	7.0 (6.0, 8.0)	7.0 (6.0, 8.0)	8.0 (6.0, 8.0)	7.0 (6.0, 8.0)	< 0.001^*^
**OPA (MET-h/day)**	19.9 (11.9, 31.1)	21.1 (10.6, 42.3)	36.3 (20.2, 59.4)	17.3 (10.7, 24.6)	31.7 (20.1, 45.2)	< 0.001^*^
**TPA,** ***n*** **(%)**	7270 (95.0)	986 (91.8)	379(90.9)	4500 (96.3)	1405 (94.5)	< 0.001^**^
**DPA,** ***n*** **(%)**	7265 (95.0)	1012 (94.2)	391 (93.8)	4471 95.7)	1391 (93.5)	0.002^**^
**LTPA,** ***n*** **(%)**	915 (12.0)	158 (14.7)	90 (21.6)	456 (9.8)	211 (14.2)	< 0.001^**^
**Men,** ***n*** **(%)**	2798 (36.6)	310 (28.9)	195 (46.8)	1534 (32.8)	759 (51.0)	< 0.001^**^
**Married/Cohabitating,** ***n*** **(%)**	6900 (90.2)	885 (82.4)	389 (92.6)	4230 (90.6)	1399 (94.1)	< 0.001^**^
**Smoking,** ***n*** **(%)**	1423 (18.6)	168 (15.6)	93 (22.3)	800 (17.1)	362 (24.3)	< 0.001^**^
**Alcohol drinking,** ***n*** **(%)**	3643 (47.6)	473 (44.0)	263 (63.1)	1995 (42.7)	912 (61.3)	< 0.001^**^
**Tea intake,** ***n*** **(%)**	992 (13.0)	142 (13.2)	66 (15.8)	586 (12.5)	198 (13.3)	0.264^**^
**Beverage intake,** ***n*** **(%)**	260 (3.4)	35 (3.3)	21 (5.0)	131 (2.8)	73 (4.9)	< 0.001^**^
**Ethnic,** ***n*** **(%)**						< 0.001^**^
** Miao**	1990 (26.0)	349 (32.5)	138 (33.1)	1114 (23.8)	389 (26.2)	
** Dong**	3283 (42.9)	405 (37.7)	138 (33.1)	2136 (45.7)	604 (40.6)	
** Bouyei**	2376 (31.1)	320 (29.8)	141 (33.8)	1421 (30.4)	494 (33.2)	
**Highest education completed,** ***n*** **(%)**						< 0.001^**^
** No formal school**	4442 (58.1)	695 (64.7)	124 (29.7)	3079 (65.9)	544 (36.6)	
** Primary school**	1502 (19.6)	175 (16.3)	99 (23.7)	881 (18.9)	347 (23.3)	
** Middle school**	1263 (16.5)	143 (13.3)	101 (24.2)	600 (12.8)	419 (28.2)	
** High school**	290 (3.8)	41 (3.8)	48 (11.5)	103 (2.2)	98 (6.6)	
** College or university**	152 (2.0)	20 (1.9)	45 (10.8)	8 (0.2)	79 (5.3)	
**Annual income, whole family (Yuan/year),** ***n*** **(%)**						< 0.001^**^
** < 12 000**	3191 (41.7)	480 (44.7)	88 (21.1)	2176 (46.6)	447 (30.1)	
** 12 000 − 19 999**	1971 (25.8)	211 (19.6)	86 (20.6)	1250 (26.8)	424 (28.5)	
** 20 000 − 59 999**	1935 (25.3)	280 (26.1)	153 (36.7)	1032 (22.1)	470 (31.6)	
** 60 000 − 99 999**	359 (4.7)	67 (6.2)	53 (12.7)	136 (2.9)	103 (6.9)	
** ≥ 100 000**	188 (2.5)	35 (3.3)	37 (8.9)	74 (1.6)	42 (2.8)	

Data are presented as numbers (percentage) or medians (interquartile range). ^*^Kruskal–Wallis test; ^**^Chi-square test. *BMI* Body mass index, *TC* Total cholesterol, *TG* Triglycerides, *HDL-C* High-density lipoprotein-cholesterol, *LDL-C* Low-density lipoprotein-cholesterol, *OPA* Occupational physical activity, *TPA* Transportation physical activity, *DPA* Domestic physical activity, *LTPA* Leisure-time physical activity

### Serum lipid and OPA levels

**Fig. 2:**
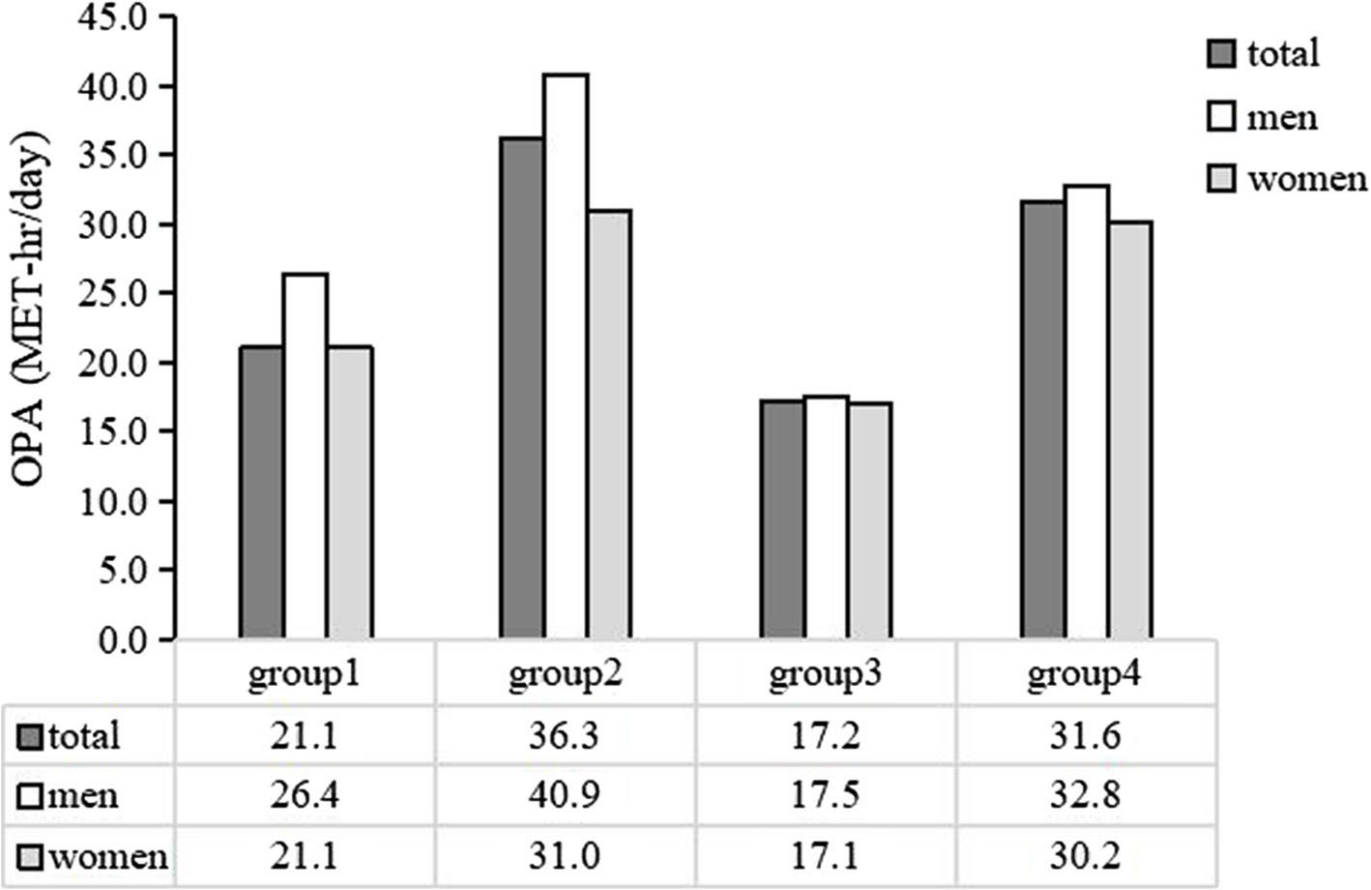
Occupational physical activity (OPA) level according to the working mode categories. The group1, group2, group3, and group4 indicate no change, changing job, seasonal changes, and seasonal and job changes, respectively

Figure 2 shows the OPA levels in each working mode group. In each group, men had higher OPA levels than women. People with other jobs had a high level of OPA, regardless of seasonal changes in their farming work.

**Fig. 3:**
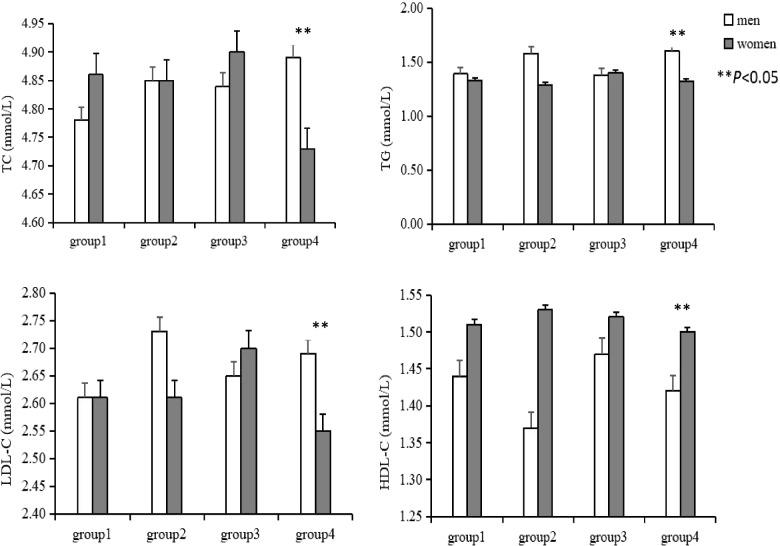
Each serum lipid level according to the working mode categories. The group1, group2, group3, and group4 indicate no change, changing job, seasonal changes, and seasonal and job changes, respectively. TC, total cholesterol; TG, triglyceride; LDL-C, low-density lipoprotein cholesterol; HDL-C, high-density lipoprotein cholesterol

Figure 3 shows the serum lipid levels in each working mode group according to sex. In working mode 4, men had higher levels of TG, TC, and LDL-C and lower levels of HDL-C than women (all *P* values < 0.05).

### Association between OPA in different working mode groups and dyslipidaemia

Table 2 shows the associations between OPA and dyslipidaemia in different groups. Q1 is the comparison group. After adjusting for the potentially confounding effects of age, education, sex, smoking, drinking, leisure sedentary time, sleeping time, total energy intake, married or cohabitating, beverage intake, TPA, DPA, LTPA, BMI, ethnicity, and annual income, the logistic regression analysis revealed that in the no change group, the Q2–Q3 or Q4 level of OPA was associated with a low risk of high TC (Q2–Q3: adjusted OR = 0.56, 95% CI: 0.34–0.91; Q4: adjusted OR = 0.41, 95% CI: 0.18–0.96) and LDL-C (Q2–Q3: adjusted OR = 0.51, 95% CI: 0.28–0.92). In the seasonal change group, the Q4 level of OPA was associated with a low risk of high TC (Q4: adjusted OR = 0.74, 95% CI: 0.55–0.99), low HDL-C (Q4: adjusted OR = 0.64, 95% CI: 0.44–0.92), and high LDL-C (Q4: adjusted OR = 0.63, 95% CI: 0.43–0.92). In the seasonal and job change group, the Q2–Q3 level of OPA could increase the risk of high TC (Q2–Q3: adjusted OR = 2.15, 95% CI: 1.25–3.69).

**Table 2:** Relationship between occupational physical activity and dyslipidaemia in the total population with different working modes

Working modes	TC ≥ 6.22 mmol/L	TG ≥ 2.26 mmol/L	HDL–C < 1.04 mmol/L	LDL–C ≥ 4.14 mmol/L
	**OR (95% CI)**	**OR (95% CI)**	**OR (95% CI)**	**OR (95% CI)**
**No change**				
** Q1**	Reference	Reference	Reference	Reference
** Q2–Q3**	0.56 (0.34––0.91)^*^	0.89 (0.63–1.26)	1.14 (0.61–2.12)	0.51 (0.28–0.92)^*^
** Q4**	0.41 (0.18––0.96)^*^	0.60 (0.35–1.04)	0.32 (0.09–1.15)	0.63 (0.26–1.53)
**Changing job**				
** Q1**	Reference	Reference	Reference	Reference
** Q2–Q3**	0.60 (0.24––1.51)	0.95 (0.49–1.81)	1.16 (0.36–3.73)	1.19 (0.29–4.89)
** Q4**	0.93 (0.32––2.78)	0.95 (0.43–2.13)	1.11 (0.26–4.81)	1.64 (0.31–8.66)
**Seasonal changes**				
** Q1**	Reference	Reference	Reference	Reference
** Q2–Q3**	0.93 (0.73––1.19)	1.02 (0.85–1.22)	0.89 (0.67–1.19)	0.99 (0.74–1.32)
** Q4**	0.74 (0.55–0.99)^*^	0.81 (0.66–1.01)	0.64 (0.44–0.92)^*^	0.63 (0.43–0.92)^*^
**Seasonal and job changes**				
** Q1**	Reference	Reference	Reference	Reference
** Q2–Q3**	2.15 (1.25–3.69)^*^	0.79 (0.58–1.08)	0.50 (0.32–1.79)	2.07 (0.59–4.06)
** Q4**	1.52 (0.81–2.85)	0.73 (0.51–1.05)	0.55 (0.32–1.94)	0.72 (0.29–1.83)

All odds ratios were calculated relative to the Q1 level of occupational physical activity and were adjusted for age, education, sex, smoking, drinking, leisure sedentary time, sleeping time, BMI, total energy intake, married or cohabitating, beverage intake, TPA, DPA, LTPA, ethnicity, and annual income. *OR* Odds ratio, *CI* Confidence interval, *TC* Total cholesterol, *TG* Triglycerides, *HDL-C* High-density lipoprotein-cholesterol, *LDL-C* Low-density lipoprotein-cholesterol, Q1, OPA ≤ P_25_; Q2, P_25_ < OPA < P_75_; Q3, OPA ≥ P_75_. ^*^P value < 0.05

Table 3 shows the associations between OPA and dyslipidaemia in different working modes in men. Q1 is the comparison group. The adjusted variables are consistent with those present in Table 2. In the no change group, the Q2–Q3 or Q4 level of OPA was associated with a low risk of high TC (Q2–Q3: adjusted OR = 0.21, 95% CI: 0.07–0.64; Q4: adjusted OR = 0.04, 95% CI: 0.01–0.40). In the seasonal change group, the Q4 level of OPA was associated with a low risk of low HDL-C (Q4: adjusted OR = 0.46, 95% CI: 0.27–0.77). In the seasonal and job change group, the Q2–Q3 level of OPA could increase the risk of high LDL-C (Q2–Q3: adjusted OR = 3.23, 95% CI: 1.06–9.80).

**Table 3:** Relationship between occupational physical activity and dyslipidaemia in men with different working modes

Working modes	TC ≥ 6.22 mmol/L	TG ≥ 2.26 mmol/L	HDL–C < 1.04 mmol/L	LDL–C ≥ 4.14 mmol/L
	**OR (95% CI)**	**OR (95% CI)**	**OR (95% CI)**	**OR (95% CI)**
**No change**				
** Q1**	Reference	Reference	Reference	Reference
** Q2–Q3**	0.21 (0.07–0.64)^*^	0.69 (0.35–1.36)	0.69 (0.26–1.85)	0.57 (0.19–1.68)
** Q4**	0.04 (0.01–0.40)^*^	0.47 (0.18–1.21)	0.27 (0.05–1.43)	0.21 (0.03–1.32)
**Changing job**				
** Q1**	Reference	Reference	Reference	Reference
** Q2–Q3**	0.89 (0.18–4.43)	0.92 (0.37–2.29)	0.33 (0.07–1.68)	3.03 (0.14–64.22)
** Q4**	1.26 (0.21–7.59)	0.47 (0.15–1.41)	0.19 (0.02–1.54)	2.76 (0.14–54.52)
**Seasonal changes**				
** Q1**	Reference	Reference	Reference	Reference
** Q2–Q3**	1.10 (0.70–1.74)	0.91 (0.67–1.24)	0.70 (0.46–1.05)	0.89 (0.53–1.48)
** Q4**	1.15 (0.69–1.93)	0.71 (0.490–1.02)	0.46 (0.27–0.77)^*^	0.63 (0.33–1.10)
**Seasonal and job changes**				
** Q1**	Reference	Reference	Reference	Reference
** Q2–Q3**	1.70 (0.81–3.57)	0.77 (0.52–1.15)	0.39 (0.22–1.70)	3.23 (1.06–9.80)^*^
** Q4**	1.51 (0.65–3.51)	0.67 (0.42–1.07)	0.54 (0.28–1.02)	1.31 (0.33–5.17)

All odds ratios were calculated relative to the Q1 level of occupational physical activity and were adjusted for age, education, sex, smoking, drinking, leisure sedentary time, sleeping time, BMI, total energy intake, married or cohabitating, beverage intake, TPA, DPA, LTPA, ethnicity, and annual income. *OR* Odds ratio, *CI* Confidence interval, *TC* Total cholesterol, *TG* Triglycerides, *HDL-C* High-density lipoprotein-cholesterol, *LDL-C* Low-density lipoprotein-cholesterol, Q1, OPA ≤ P_25_; Q2, P_25_ < OPA < P_75_; Q3, OPA ≥ P_75_. ^*^P value < 0.05

Table 4 shows the associations between OPA and dyslipidaemia in different working modes in women. The adjusted variables are consistent with those presented in Table 2. In the no change group, the Q2–Q3 level of OPA was associated with a low risk of high LDL-C (Q2–Q3: adjusted OR = 0.43, 95% CI: 0.20–0.93). In the seasonal change group, the Q4 level of OPA was associated with a low risk of high TC (Q4: adjusted OR = 0.59, 95% CI: 0.41–0.86). In the seasonal and job change group, the Q2–Q3 level of OPA could increase the risk of high TC (Q2–Q3: adjusted OR = 3.24, 95% CI: 1.42–7.41).

**Table 4:** Relationship between occupational physical activity and dyslipidaemia in women with different working modes

Working modes	TC ≥ 6.22 mmol/L	TG ≥ 2.26 mmol/L	HDL–C < 1.04 mmol/L	LDL–C ≥ 4.14 mmol/L
	**OR (95% CI)**	**OR (95% CI)**	**OR (95% CI)**	**OR (95% CI)**
**No change**				
** Q1**	Reference	Reference	Reference	Reference
** Q2–Q3**	0.71 (0.40–1.27)	0.99 (0.65–1.51)	1.65 (0.69–3.94)	0.43 (0.20–0.93)^*^
** Q4**	0.76 (0.30–1.91)	0.67 (0.33–1.36)	0.31 (0.04–2.63)	1.08 (0.37–3.11)
**Changing job**				
** Q1**	Reference	Reference	Reference	Reference
** Q2–Q3**	0.61 (0.17–2.20)	1.11 (0.38–3.28)	6.52 (0.62–68.95)	1.08 (0.12–9.87)
** Q4**	0.58 (0.10–3.26)	3.28 (0.92–11.72)	5.97 (0.36–98.12)	1.31 (0.10–17.32)
**Seasonal changes**				
** Q1**	Reference	Reference	Reference	Reference
** Q2–Q3**	0.85 (0.64–1.14)	1.08 (0.85–1.36)	1.12 (0.73–1.70)	1.10 (0.71–1.45)
** Q4**	0.59 (0.41–0.86)^*^	0.89 (0.67–1.18)	0.88 (0.52–1.49)	0.63 (0.39–1.00)
**Seasonal and job changes**				
** Q1**	Reference	Reference	Reference	Reference
** Q2–Q3**	3.24 (1.42–7.41)^*^	0.88 (0.54–1.45)	0.80 (0.36–1.67)	1.93 (0.79–4.75)
** Q4**	1.79 (0.66–4.84)	0.87 (0.47–1.58)	0.51 (0.17–1.52)	0.50 (0.12–2.02)

All odds ratios were calculated relative to the Q1 level of occupational physical activity and were adjusted for age, education, sex, smoking, drinking, leisure sedentary time, sleeping time, BMI, total energy intake, married or cohabitating, beverage intake, TPA, DPA, LTPA, ethnicity, and annual income. *OR* Odds ratio, *CI* Confidence interval, *TC* Total cholesterol, *TG* Triglycerides, *HDL-C* High-density lipoprotein-cholesterol, *LDL-C* Low-density lipoprotein-cholesterol, Q1, OPA ≤ P_25_; Q2, P_25_ < OPA < P_75_; Q3, OPA ≥ P_75_. ^*^P value < 0.05

## Discussion

In the present large population-based study of Chinese farmers, OPA with different work patterns exhibited differences in the association with varying blood lipid levels. In the no change and seasonal change groups, the level of OPA was low, and active OPA reduced the risk of dyslipidaemia. In the changing job and seasonal and job change groups, the level of OPA was high; however, active OPA increased the risk of dyslipidaemia only in the seasonal and job change group. No association was observed between any level of OPA and any type of dyslipidaemia in the changing job group.

In terms of population characteristics and OPA, generally on an average, in the changing job, seasonal change, and seasonal and job change groups, younger men (more likely to be married men) had lower education level and annual income and a higher level of total energy intake. In addition, they tended to smoke and drink. This finding is consistent with the actual situation and those of some previous studies [18, 19]. Generally, most farmers’ education and family income were not high; hence, they were engaged in manual labour. Traditionally, men work in the fields, and women are mostly responsible for housework [20]. Additionally, age limited the farmers’ mode of agricultural production. Some young farmers chose to engage in other jobs after finishing their agricultural work to support their family. However, elderly farmers engage less in agricultural activities because of their low physical fitness and coordination ability. LTPA is also crucial to human health. However, this study observed a low LTPA participation rate of the farmers, and the finding is consistent with those of previous studies [21–23]. One possible explanation is that OPA might be an obstacle to LTPA [24, 25], even among farmers.

Previous studies have reported that PA could reduce the risk of dyslipidaemia but had weak association with LDL-C or TC [9, 26, 27]. However, in this study, we divided farmers into four groups according to different working modes and observed that in the no change and seasonal change groups, active OPA could reduce the risk of dyslipidaemia, mainly by reducing the TC or LDL-C level. Specifically, in the no change and seasonal change groups, the Q2–Q3 and Q4 levels of OPA were associated with a low risk of high LDL-C in the total population. In the no change group, the Q2–Q3 level of OPA was associated with a low risk of high LDL-C in women. However, in the seasonal and job change group, we observed a special phenomenon; the Q2–Q3 level of OPA could increase the risk of high LDL-C in men. LDL-C is the key marker related to atherosclerosis. We tried to explain it with seasonality. Studies have reported that among the blood lipid indicators, TC and LDL-C indices are the most sensitive to seasonal changes, and the working modes may vary across different seasons [28]. The findings of Zhou X [29] and Hadaegh F [30] have shown that LDL-C or TC levels increase in winter and decrease in summer. Seasonal changes in related factors, such as moderate OPA and farmers' particular work modes, can be used to explain a part of the observed seasonal changes in lipid levels [31]. In general, for farmers' production activities, spring and winter are non-farming seasons, while summer and autumn are farming seasons. In the farming season, to prevent any delay in the farming processes and failure in working in the good farming weather, farmers might engage in high-intensity physical labour for a long time [13]. If farmers have to do additional work after busy farm work in this time duration, the level of OPA accordingly increases, which may have an adverse impact on the level of blood lipids. OPA leads to changes in the blood volume in different working modes. Thus, in the no change and seasonal change groups, the high levels of OPA might reduce the risk of high TC or high LDL-C. In the seasonal and job change groups, the high levels of OPA might increase the risk of high TC or high LDL-C [31]. However, prospective research must be performed for further exploration. Our results suggested that we should pay more attention to the OPA level of men with seasonal and job changes in the future because these changes may increase the risk of atherosclerosis in them. Unlike those in previous studies, participants in this study were farmers. The nature and mode of their work are not consistent with those of other occupational groups. In addition, genes, living environment, and eating habits [32] might be some of the major factors for differences between the results of the present study and those of other studies, warranting further investigation.

Additionally, the OPA level was high in people who reported other jobs regardless of seasonal changes in farming work, indicating that engagement in other jobs might increase the OPA level. In the seasonal and job change group, men had higher levels of TG, TC, and LDL-C and lower levels of HDL-C than women, and the Q2–Q3 level of OPA could increase the risk of dyslipidaemia. Holtermann A et al. reported six reasons for the ineffectiveness of OPA in improving cardiovascular health like LTPA [33]. We speculate that this may be related to the working mode and work intensity of farmers. It is well-documented that in farming seasons, farmers often need more than 10 h of OPA for a continuous time period on a daily basis, and the frequency and time of rest after work are limited. Repeated, monotonous, and high-intensity OPA for a long time (such as transplanting, ploughing, and harvesting rice) lead to an increase in the heart rate, and the increased heart rate for a long time is an independent risk factor for CVD-related diseases [34]. Studies have reported that high-intensity OPA for > 8 h a day can adversely affect cardiovascular health [35]. In addition, in this working mode, some people engage in other OPA (as a builder or porter) in addition to agricultural activities. These additional OPA may increase their physical and psychological stress, providing insufficient time for recovery, which eventually leads to overwork and continuous increase in inflammatory marker levels, finally increasing the risk of atherosclerosis [36].

Although the development of global economy has brought the era of mechanisation into the life of farmers [37], most areas of Guizhou Province have not yet achieved comprehensive mechanised work because of the limitation of geographical environment. Therefore, most of the agricultural production activities of farmers still rely on the traditional manual or semi-mechanical production. The semi-mechanical production mode only shortens the time of traditional manual farming but does not markedly reduce physical labour of farmers. This has made most farmers, especially those under economic distress, engage in some other work after finishing farming in the farming season. Undoubtedly, the amount and intensity of PA would be too high, exceeding body’s own bearing capacity, thereby affecting body’s regulatory function and resulting in stress and stress tolerance imbalance [38]. Ultimately, the risk of dyslipidaemia increases. The reason for no association between the Q4 level of OPA and dyslipidaemia in the seasonal and job change group might be the small sample size of the present study. Additionally, although the level of OPA was high in the changing job group, our results revealed no correlation between OPA at any level and dyslipidaemia in this group. The large number of men might be one of the reasons for the high level of PA in this group because men were always engaged in heavy work-related PA, and the small sample size might be an important reason for the lack of statistical significance between OPA and dyslipidaemia in this group.

This study has important implications for public health. Exercise can improve body’s metabolism and promote mental health. The present data suggest the necessity to develop OPA guidelines for farmers. Moderate OPA may help in reducing the risk of dyslipidaemia in a primary care setting or a large cohort. Farmers with seasonal changes in farming work and those involved in other jobs should carefully manage their OPA level. Considering the ongoing dyslipidaemia epidemics affecting contemporary society, understanding the association of OPA in different working modes with dyslipidaemia among farmers is of great importance for public health.

This study presents several advantages. First, to the best of our knowledge, this study is the first large-scale epidemiological study conducted by a Chinese group on ethnic minorities in Southwest China by using a suitable electronic questionnaire to investigate the OPA characteristics of farmers and preliminarily explored their relationship with dyslipidaemia, which may be useful for further research. Second, it combined the characteristics of current farmers’ working modes, divided the farmers into four different characteristic groups, and discussed the relationship between OPA and dyslipidaemia under different working modes. To our knowledge, this study is the first in southwest China to link OPA with dyslipidaemia among farmers, which holds reference significance in clinical and public health.

This study also has some limitations. First, OPA in our questionnaire was self-reported. Second, because of the cross-sectional study design, causal inference was limited and could only provide clues about aetiology, warranting further follow-up studies. Finally, the influence of genetic factors could not be addressed in the present study.

## Conclusion

This study suggested that different OPA levels might exhibit different associations with blood lipid levels. According to our results, in the no change and seasonal change groups, the active OPA reduced the risk of dyslipidaemia. In the seasonal and job change groups, active OPA led to the increase in the risk of dyslipidaemia. Therefore, in the no change and seasonal change groups, the working mode of OPA was feasible. In addition, this study showed that the OPA guidelines vary for farmers with different working modes. In particular, our research suggested that farmers in the seasonal and job change groups should try to reduce the level of OPA. We will continue to study the relationship between OPA and blood lipids in this population and explore OPA conducive to maintaining a high blood lipid level.

## Supplementary Information

**Additional file 1: Figure S1.** Directed acyclic graphs adjusting for confounders.

**Additional file 2: Supplemental Material S1.** The China Multi-Ethnic Cohort (CMEC) Study questionnaire on occupational physical activity among non-farmers and farmers. **Supplemental Material S2.** The measurement methods for weight and height. **Supplemental Table S1.** Occupational physical activity types, MET values, and intensity categories.

## Data Availability

Currently, the databases used to support this study are not freely available in view of participants’ privacy protection but are available from the corresponding author on reasonable data request. Researchers interested in our study could contact the corresponding author Dr. Feng Hong (519,490,967@qq.com) who will review the data request.

## Abbreviations

BMI: Body mass index
CMEC: China Multi-Ethnic Cohort
CKB: China Kadoorie Biobank
DAG: Directed acyclic graph
DPA: Domestic physical activity
HDL-C: High-density lipoprotein-cholesterol
LDL-C: Low-density lipoprotein-cholesterol
LTPA: Leisure-time physical activity
METs: Metabolic equivalent tasks
OPA: Occupational physical activity
ORs: Odds ratio
PA: Physical activity
TC: Total cholesterol
TG: Triglycerides
TPA: Transportation physical activity
95% CIs: Confidence intervals
