# Supplementary figures and images for: The relationship between occupational physical activity and dyslipidaemia in farmers with varying working modes in southwest China: the China multi-ethnic cohort study
Source: BMC Public Health. 2022 Apr 27;22:840. doi: 10.1186/s12889-022-13266-x (PMC9044674; doi:10.1186/s12889-022-13266-x)

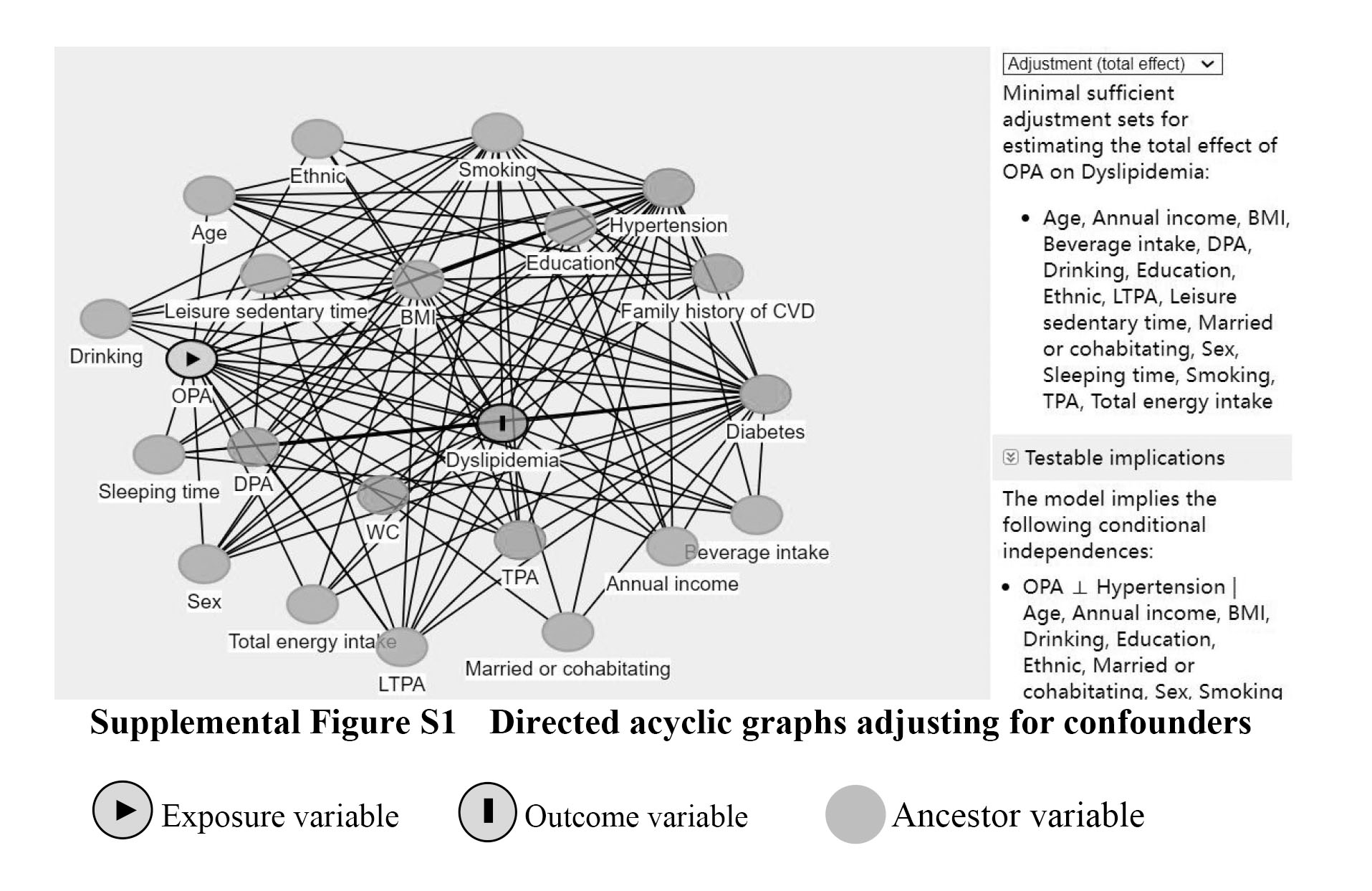

Supplement: Supplementary file 1 — Additional file 1: Figure S1. Directed acyclic graphs adjusting for confounders. [file 12889_2022_13266_MOESM1_ESM.jpg]
