# Supplementary material for: The relationship between occupational physical activity and dyslipidaemia in farmers with varying working modes in southwest China: the China multi-ethnic cohort study
Source: BMC Public Health. 2022 Apr 27;22:840. doi: 10.1186/s12889-022-13266-x (PMC9044674; doi:10.1186/s12889-022-13266-x)
Supplement: Supplementary file 2 — Additional file 2: Supplemental Material S1. The China Multi-Ethnic Cohort (CMEC) Study questionnaire on occupational physical activity among non-farmers and farmers. Supplemental Material S2. The measurement methods for weight and height. Supplemental Table S1. Occupational physical activity types, MET values, and intensity categories. [file 12889_2022_13266_MOESM2_ESM.doc]

# Supplemental Materials

# Supplemental Material S1. The China Multi-Ethnic Cohort (CMEC) Study questionnaire on occupational physical activity among non-farmers and farmers

## Section A: For non-farmers

1. In the past 12 months, how active were you at work?

🞎 Mainly sedentary (e.g. office worker, secretary) 🡪sitting time hrs/week

🞎 Standing occupation (e.g. guard, shop assistant)

🞎 Manual work (e.g. plumber, carpenter)

🞎 Heavy manual work (e.g. miner, construction worker)

🞎 Retired, housewife/husband, unemployed, or disabled

## Section B: For farmers

1. In the past 12 months, did your farming work change seasonally?

🞎 Yes

🞎 No 🡪  *go to Question 6*

In the farming season in the last 12 months:

2. How many months did the farming season usually last? months

3. What types of farming work did it usually involve?

🞎 Manual 🞎 Semi-mechanized 🞎 Fully mechanized

4. How many hours did you usually work each day? hrs

5. Of which, how many hours did you sweat or have a much faster heartbeat? hrs

6. In a typical week (in non-farming seasons), How much time do you do farm work on average per week? hrs/week

7. Apart from the agriculture work, did you have any other job?

🞎 Yes

🞎 No 🡪 *go to Question transportation physical activity^*^*

8. How active were you at work with the other job?

🞎 Mainly sedentary 🡪 sitting time hrs/week 🞎 Mainly general manual work

🞎 Mainly standing 🞎 Mainly heavy manual work

9. In a typical week, about how many hours did you spent at the other job? Hrs

^*^Because this study mainly discusses OPA, only the questions related to OPA are listed in the Supplemental Material , and other questions related to other physical activity are not listed.

**Supplemental Material S2.** The measurement methods for weight and height.

1. The measurement methods for weight

The weight is measured by electronic weight meter (unit: kg). When measuring, place the weight meter on the horizontal ground stably. Ask the participants to take off his hat, coat and shoes, only wear single-layer clothes, take out his belongings and stand in the center of the scale plate. After the display value of the scale is stable, fill the weight results in the corresponding position of the physical examination form, and the measurement results are accurate to 0.1kg.

1. The measurement methods for height

Ultrasonic height measuring instrument (unit: cm) is adopted. During the measurement, the participants were asked to wear light clothes, take off their hats and barefoot. The head is upright, the trunk is naturally straight, the eyes are level, the upper limbs sag from the disc, the legs are straight, the heels of the feet are close together, and the toes are separated by about 60°. The heel, sacrum and two shoulder blades are in contact with the wall to form a "three-point and one-line" standing posture. After pressing the button of ultrasonic height measuring instrument, ask the respondents to leave the measurement range to avoid reading interference. Press the button of the ultrasonic height measuring instrument again. After hearing the dropping sound twice, record the height reading, and the measurement result is accurate to 0.1cm.

**Supplemental Table S1.** Occupational physical activity types, MET values, and intensity categories^**^

| **Activity type** | **Intensity** | **MET** |
| --- | --- | --- |
| **Non-farmers** |  |  |
| Heavy manual work | Vigorous | 6.5 |
| Manual work | Moderate | 4.5 |
| Standing work | Moderate | 3.8 |
| Sedentary work | Low | 1.8 |
| **Farmers** |  |  |
| Manual work in the farming season | Vigorous | 6.3 |
| Semi-mechanized work in the farming season | Moderate | 3.4 |
| Fully mechanized work in the farming season | Low | 2.4 |
| Work outside the farming season | Low | 2.0 |

MET: Metabolic equivalent of tasks. ^**^ Based on the 2011 Compendium of Physical Activities: a second update of codes and MET values. Ainsworth BE, et al. Medicine and Science in Sports and Exercise, 2011;43(8):1575-1581.
